# Opposed Actions of PKA Isozymes (RI and RII) and PKC Isoforms (cPKCβI and nPKCε) in Neuromuscular Developmental Synapse Elimination

**DOI:** 10.3390/cells8111304

**Published:** 2019-10-23

**Authors:** Neus Garcia, Cori Balañà, Maria A. Lanuza, Marta Tomàs, Víctor Cilleros-Mañé, Laia Just-Borràs, Josep Tomàs

**Affiliations:** Unitat d’Histologia i Neurobiologia (UHNEUROB), Facultat de Medicina i Ciències de la Salut, Universitat Rovira i Virgili. Sant Llorenç 21, 43201 Reus, Spain; coribm.1991@gmail.com (C.B.); mariaangel.lanuza@urv.cat (M.A.L.); marta.tomas@urv.cat (M.T.); victorcilleros@gmail.com (V.C.-M.); l.just.borras@gmail.com (L.J.-B.)

**Keywords:** motor end-plate, postnatal synapse elimination, acetylcholine release, muscarinic acetylcholine receptors, PKC, PKA, TrkB receptors, adenosine receptors

## Abstract

Background: During neuromuscular junction (NMJ) development, synapses are produced in excess. By sensing the activity-dependent release of ACh, adenosine, and neurotrophins, presynaptic receptors prompt axonal competition and loss of the unnecessary axons. The receptor action is mediated by synergistic and antagonistic relations when they couple to downstream kinases (mainly protein kinases A and C (PKA and PKC)), which phosphorylate targets involved in axonal disconnection. Here, we directly investigated the involvement of PKA subunits and PKC isoforms in synapse elimination. Methods: Selective PKA and PKC peptide modulators were applied daily to the *Levator auris longus* (LAL) muscle surface of P5–P8 transgenic B6.Cg-Tg (Thy1-YFP) 16 Jrs/J (and also C57BL/6J) mice, and the number of axons and the postsynaptic receptor cluster morphology were evaluated in P9 NMJ. Results: PKA (PKA-I and PKA-II isozymes) acts at the pre- and postsynaptic sites to delay both axonal elimination and nAChR cluster differentiation, PKC activity promotes both axonal loss (a cPKCβI and nPKCε isoform action), and postsynaptic nAChR cluster maturation (a possible role for PKCθ). Moreover, PKC-induced changes in axon number indirectly influence postsynaptic maturation. Conclusions: PKC and PKA have opposed actions, which suggests that changes in the balance of these kinases may play a major role in the mechanism of developmental synapse elimination.

## 1. Introduction

During the histogenesis of the nervous system, both neurons and synapses are produced in excess. Subsequent refinement occurs throughout the nervous system [1,2,3] and leads to maturation, improves function, and ensures that the appropriated connections remain, whereas the redundant ones are eliminated [4,5,6].

At birth, neuromuscular junctions (NMJs) are polyinnervated but, by the end of axonal competition, the motor endplates are innervated by only one axon [6,7,8,9,10,11]. Activity-dependent signaling through membrane receptors enables axon terminals and synapses to influence each other directly or through the postsynaptic component and neighbor glial cells [12,13,14,15]. Presynaptic muscarinic ACh autoreceptors (mAChR; M_1_, M_2_, and M_4_ subtypes), adenosine receptors (AR; A_1_ and A_2A_), and the tropomyosin-related kinase B receptor (TrkB) cooperate in promoting axonal competition during the first 2 weeks after birth [16,17], and also NMDA glutamate receptors take part in this process [18,19]. In previous studies we investigated the synergistic and antagonistic relations between these receptors that affect synapse elimination [16,17].

Downstream of the membrane receptors, effector kinases regulate most cellular functions and phosphorylate the targets that mediate axonal competition and loss. M_1_ and TrkB operate mainly through the phospholipase C gamma (PLCγ)—and therefore the protein kinase C (PKC) pathways and the inositol triphosphate (IP3) pathway—whereas A_2A_, M_2_, and M_4_ are coupled to the adenylyl cyclase (AC) and the protein kinase A (PKA) pathway [20,21,22,23,24]. Other receptors such as A_1_ are negatively coupled to the AC signal-transduction pathway (through a Gi protein) and signal via phospholipase C (through a pertussis toxin sensitive Go protein) [25,26]. Additionally, G protein-coupled receptors can directly modify effector targets rather than acting through a downstream soluble intracellular messenger [27,28].

We hypothesize that the relationship between PKA and PKC activities in some nerve endings play a leading role in synapse elimination [29]. The results here show that PKA activity delays both axonal elimination and the differentiation of nicotinic acetylcholine receptor (nAChR) clusters. Contrarily, PKC activity promotes both axonal loss and postsynaptic maturation with the involvement of presynaptic cPKCβI and nPKCε and maybe postsynaptic PKCθ isoforms. Therefore, the effect of blocking a PKC isoform is similar to that of PKA stimulation, which emphasizes the functional relevance of the appropriate balance of both kinases activity. We also found a clear indication that axonal loss indirectly influences postsynaptic maturation.

## 2. Materials and Methods

### 2.1. Animals

Two mice strains were used: B6.Cg-Tg (Thy1-YFP) 16Jrs/J (Thy1-YFP-16) and C57BL/6J (wild type control) from the Jackson Laboratory. Thy1-YFP-16 mice express yellow fluorescent protein driven by a mouse *Thy1* promoter, which labels sensory and motor neurons as well as subsets of central neurons. This line provides a strong and specific vital marker for axons. No expression is detectable in nonneural cells. All experiments were conducted on Thy1-YFP expressing mice. In some cases, we checked our results with C57BL/6J mice and found no significant differences with YFP mice. Procedures, mice care, and experimental protocols were performed according the guidelines of the European Community’s Council Directive of 24 November 1986 (86/609/EEC) for the humane treatment of laboratory animals. Animal Research Committee of the Universitat Rovira i Virgili (Reference number: 0233) reviewed and approved all experiments on animals.

All animals were neonatal pups of either sex. The date of birth was designated postnatal day 0 (P0). We minimized the variability in our measurements by carefully monitoring the timing of conception and the weights of the individuals at P9, which were within ±5% of the mean. Whole *Levator auris longus* (LAL) muscles were used to perform the morphological analysis at postnatal day 9.

### 2.2. Injection Procedure

The newborn mice were treated once a day from P5 to P8. Previously to manipulation, the animals were anesthetized with diethyl ether (Merk, Kenilworth, NJ, USA) via inhalation. Under aseptic conditions, the treatments were administered by subcutaneous injections over the LAL muscle, in the back of the neck as previously described [17,30]. Antagonists and agonists were diluted to the convenient concentration in phosphate-buffered saline (PBS), and 50 μL were injected from P5 to P8 or from P5 to P14. The experimental modulation of the molecular pathways related with developmental axonal competition and loss at P5–P9 and observation of the result at P9 is a good strategy to acquire a broad information of the mechanisms involved.

Control experiments were done by injection with PBS and DMSO (Sigma-Aldrich, Saint Louis, MO, USA) over the LAL muscle. The muscles injected with PBS did not show differences with the non-injected muscles in either the nAChR cluster morphology or the number of axons per endplate. The injection procedure did not induce changes in the overall morphology of the motor endplate and nerve terminals (*p* > 0.05, Fisher’s test; data not shown, see also [17]). The final concentration of DMSO in control and drug-treated preparations was 0.1% (*v*/*v*). In control experiments, the injection of 0.1% of DMSO over the LAL muscle did not affect any of the parameters studied (data not shown). The solutions were administered at a concentration in accordance with the reported biological action of the substances [31,32,33,34,35,36,37,38,39,40].

### 2.3. Tissue Preparation and Histochemistry 

Neonatal pups were given a lethal dose of 2% tribromoethanol (Sigma-Aldrich). Their heads were removed and fixed for 1.5 h in 4% paraformaldehyde (Sigma-Aldrich) and rinsed 3× in PBS. After washing, LAL muscles were dissected and post-fixed for 45 min. Next, Thy1-YFP LAL muscles were incubated (1 h at room temperature) with tetramethylrhodamine conjugated α-bungarotoxin (TRITC-α-BTX, PBS containing a 1/800 dilution of 1 µg/mL; Molecular Probes, Eugene, OR, USA). 

C57BL/6J LAL muscles were processed for immunostaining and confocal analysis. Muscles were incubated in 0.1% glycine (Sigma-Aldrich) for 12 h at 4 °C and then blocked in a solution containing 4% BSA (Sigma-Aldrich) and 0.5% Triton X-100 (Sigma-Aldrich) in PBS for 12 h at 4 °C. Presynaptic motor neuron terminals were visualized by treating muscles for 24 h at 4 °C with a primary antibody against 200-kD neurofilament protein (rabbit antibody against 200-kD neurofilament, 1:1000; Sigma-Aldrich). Tissues were rinsed 3× in PBS and treated with TRITC-α-BTX (Molecular Probes, Eugene, OR, USA) to label postsynaptic nicotinic acetylcholine receptors (nAChRs) for 45 min. The neurofilament protein was visualized by the appropriate Alexa-fluor 488 donkey anti-rabbit (1/300; Molecular Probes). As a control, the antibody specificity was tested by incubation in the absence of primary antibody. No unspecific staining was observed in the three muscles used as negative controls (not shown). Whole muscles were mounted in Mowiol (Calbiochem-Merk, Kenilworth, NJ, USA) with p-phenylenediamide (Sigma-Aldrich).

LAL muscle from C57BL/6J was processed to detect the exact localization of the PKA regulatory subunits and cPKCβI, phospho PKCβI, and nPKCε isoforms using plastic embedded semithin sections for high-resolution immunofluorescence analysis of the NMJ molecules. Briefly, LALs were processed by double immunohistochemistry, which simultaneously detected PKA regulatory subunits (RI or RII) or cPKCβI or phospho PKCβI or nPKCε isoforms with nAChRs and S100. Muscles were incubated overnight at 4 °C with the rabbit antibody against PKA Iβ reg (QR-7:sc-100414, 1:100; Santa Cruz Biotechnology, Heidelberg, Germany); rabbit antibody PKA IIβ reg (C-2:sc-376778, 1:100; Santa Cruz Biotechnology); rabbit antibody against cPKCβI (C16:sc 209, 1:100; Santa Cruz Biotechnology); rabbit antibody against nPKCε (C15:sc 214, 1:200; Santa Cruz Biotechnology); rabbit antibody against PKC βI (phospho T642) (AB75657, 1:100; Abcam, Eugene, OR, USA) and a mouse anti-S-100 antibody (1:10,000, Dako, Santa Clara, CA, USA) in 0.1% Triton X-100 plus 1% BSA. Secondary antibodies conjugated with Alexa Fluor 488 or Alexa Fluor 647 (Molecular Probes) were then added and incubated for 4 h. Postsynaptic acetylcholine receptors (nAChRs) were labeled by tetramethylrhodamine alpha-bungarotoxin (TRICT-*α*-BTX, Molecular Probes). The synaptic areas from LAL muscle were selected observing the NMJ by fluorescent microscopy. Then, muscles were dehydrated with increasing concentrations of ethanol (Panreac, Química, Castellar del Vallès, Spain) and acetone (Panreac). The tissue fragments were embedded in Spurr’s resin (Merk) and sectioned in transverse orientation. Sections 0.5 to 0.7 µm thick were cut with a Reichert Ultracut E microtome (Leica Microsystems) and stretched on glass slides by heating on a hotplate. To test the specificity of the various antibodies, we used two negative controls. One control was to omit the primary antibodies. In the second control, in double immunohistochemistry, muscles were incubated omitting either one of the two primary antibodies to show a possible cross-reaction between the primary antibodies that joined the secondary antibodies. In both cases the staining was completely removed.

### 2.4. Morphological Analysis and Confocal Microscopy 

The NMJs on LAL muscles were observed using an inverted Nikon TE-2000 fluorescent microscope (Nikon, Tokyo, Japan) connected to a personal computer running image analysis software (ACT-1, Nikon). The number of axons per endplate was counted. The NMJs were classified as junctions that were monoinnervated, doubly innervated, or innervated by three or more terminal axons. The LAL is a thin muscle which consists of only a few layers of muscle fibers. The muscle was proven to be a pure fast-twitch muscle [41]. Muscle fiber types were equally distributed in the caudal and rostral portions. NMJs from all parts of the muscle were observed and counted. NMJ with confusing superposition of the nerve terminal axons were excluded. In a reduced percentage of the triple-innervated junctions the presence of another small terminal axon can be not fully discarded (see the Figure see for exemple the 4C center, asterisk). This small percentage (typically 3–5% in different muscles) is the same in all situations tested and therefore we include these NMJ in the group of “three or more axons” synapses.

To see the effect of the treatments on the nAChR clusters, their maturation was divided into four morphological stages (S1–S4) [42,43,44]. S1: Uniform nAChR oval plaque observed at birth. S2: nAChR elongated oval plaque with some inhomogeneity in the receptor density. S3: An oval nAChR plaque with no innervated “holes” (fluorescence-free). S4: The oval nAChR areas have been changed into a mature branched pattern with convoluted external border. The edge of the holes usually has a high density of receptors. High-resolution confocal images were obtained with a 63× oil objective (1.4 numerical aperture) on a Nikon TE-2000 confocal microscope. Z stacks were obtained at step sizes of 0.5-µm for depths of 20–40 µm, and additional optical sections above and below each junction were collected to ensure that the entire synapse was included.

### 2.5. Statistical Analysis

NMJs visible in their entirety were scored, with a minimum of 100 per muscle. Twelve muscles were studied for each condition examined. Fisher’s test was applied to compare percentages. The criterion for statistical significance was *p* < 0.05. The categories were scored and the counting was performed by a person with no knowledge of the age or treatment of the animals. The data are presented as percentages of NMJ ± SD. *p* < 0.05, ** *p* < 0.01, *** *p* < 0.005.

### 2.6. Drugs 

#### 2.6.1. Selective PKC Substances

*Antagonists*: The stock solutions were Chelerytrine (CHE, C-400, Alomone, Jerusalem, Israel) 10 mM; Calfostein C (CaC, C6303, Sigma-Aldrich) 2.5 mM; Peptide βIV _5–3_ (βIV _5–3_ Mochly Rosen, Standford University) 10 mM; Peptide εV _1–2_, (εV _1–2,_ 539522. Calbiochem) 1 mM. The working solutions used were CHE (1 µM); CaC (200 nM); βIV _5–3_ (10 µM); εV _1–2_ (10 µM). 

*Agonists*: Bryostatine-1 (BRY, 2283-Totris, Minneapolis, MN, USA.) 10 µM; phorbol 12-myristate 13-acetate (PMA, P1585 Sigma) 10 mM; 12-deoxyphorbol-13-phenylacetate-20-acetate (dPPA, βI PKC selective activator, BML-PE-182-0001, Enzo Life Ccience, Farmingdale, NY, USA.) 1 mg/mL; 2-((2-Pentylcyclopropyl)methyl) cyclopropaneoctanoic acid (FR236924, ε PKC selective activator, Totris) 100 mM. The working solutions used were BRY (1 nM–10 nM); PMA (10 nM); dPPA (0.2 µg/mL); FR236924, (100 nM).

#### 2.6.2. Selective PKA Substances 

*Antagonists*: The stock solutions were dihydrochloride (H89, 19-141, Millipore-Merck) 5 mM; 8-Bromoadenosine-3′,5′-cyclic monophosphorothioate, Rp-isomer sodium salt (Rp8, RI-PKA selective, 129735-00-8, Biolog, Hayward, CA, USA) 5 mM; Adenosine-3′,5′-cyclic monophosphorothioate, Rp-isomer sodium salt (Rp, RII-PKA selective A002S, Biolog) 5 mM. The working solutions used were H89 (5 µM); Rp-8-Br-cAMPS (100–300 µM); Rp-cAMPs (100-300 µM).

*Agonist*: The stock solution was Adenosine 3’,5’-cyclic Monophosphorothioate,8-Bromo-,Sp-Isomer, Sodium Salt (Sp8Br, 116,818 Calbiochem-Merk) 5 mM. The working solution was 10 µM.

Stock solutions were prepared using PBS or DMSO in accordance with the commercial product information. All these solutions are referenced as specific but possible non-specific effects of inhibitors and stimulators can be not discarded.

### 2.7. Antibodies

Antibodies used were rabbit antibody against PKA Iβ reg (QR-7:sc-100414, Santa Cruz Biotechnology); rabbit antibody against PKA IIβ reg (C-2:sc-376778, Santa Cruz Biotechnology); rabbit antibody against cPKCβI (C16:sc 209, Santa Cruz Biotechnology); rabbit antibody against nPKCε (C15:sc 214, Santa Cruz Biotechnology); rabbit antibody against PKC βI (phospho T642) (AB75657, Abcam); and a mouse anti-S-100 antibody (Dako, Carpinteria, CA, USA).

## 3. Results

### 3.1. Postnatal Polyneuronal Innervation in the NMJ

In this study, several selective inhibitors and activators of the serine kinases PKA and PKC were subcutaneously injected over the *Levator auris longus* (LAL) mouse muscle. The developmental period P5–P9 was selected because previous studies have shown that this period is about half of the axonal loss process (the percentage of monoinnervated NMJs changes from about 20% to 60% [16]. NMJs in all stages of maturation can be observed during this period (nerve endings with different levels of transmitter release and molecular differentiation are observed), and axonal elimination is accompanied by the morphological differentiation of the postsynaptic component. During these stages, the modulation of several molecular pathways can be analyzed with different procedures to show the eventual delay or acceleration of the pre- and postsynaptic maturation.

**Figure 1:**
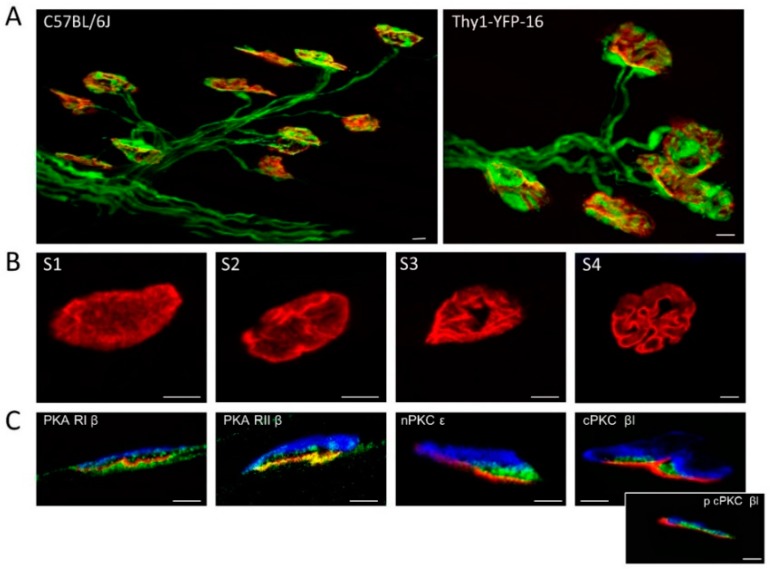
Postnatal polyneuronal innervation in the neuromuscular junctions (NMJ). In (**A**)**,** representative confocal images showing several NMJs at postnatal day 9 with different degrees of polyinnervation from C57BL/J6 mice (left, axons stained with anti neurofilament fluorescent antibody) and Thy-1-YFP-16 mice (right, autofluorescent axons). (**B**) shows the morphological maturation stages (S1–S4) of the postsynaptic nAChR clusters. In (**C**)**,** are semithin sections that show the presence of protein kinase A (PKA) regulatory subunits (RIβ and RIIβ), and nPKCε and cPKCβI (the inset shows phospho cPKCβI) isoforms in the NMJ (green). S-100 in (blue). In all cases, nAChRs are stained with tetramethylrhodamine conjugated α-bungarotoxin (red). The bars indicate 10 μm.

Figure 1A shows representative confocal fluorescence images of singly- and polyinnervated NMJs from C57BL/J6 and Thy-1-YFP-16 P9 mice. The morphological maturation (S1–S4) of the postsynaptic nAChR clusters is shown in Figure 1B. In Figure 1C are semithin sections showing the presence of PKA regulatory subunits (RIβ and RIIβ) and cPKCβI and nPKCε isoforms in the NMJ. RI and RII are located in all synaptic sites. The considered PKC isoforms were only observed with this procedure at the presynaptic site between the nAChR postsynaptic line (red) and the S-100 positive Schwann cell (blue) (see also [45]).

### 3.2. PKA Activity Modulation

#### 3.2.1. PKA Activity Prevents the Developmental Axonal Loss 

**Figure 2:**
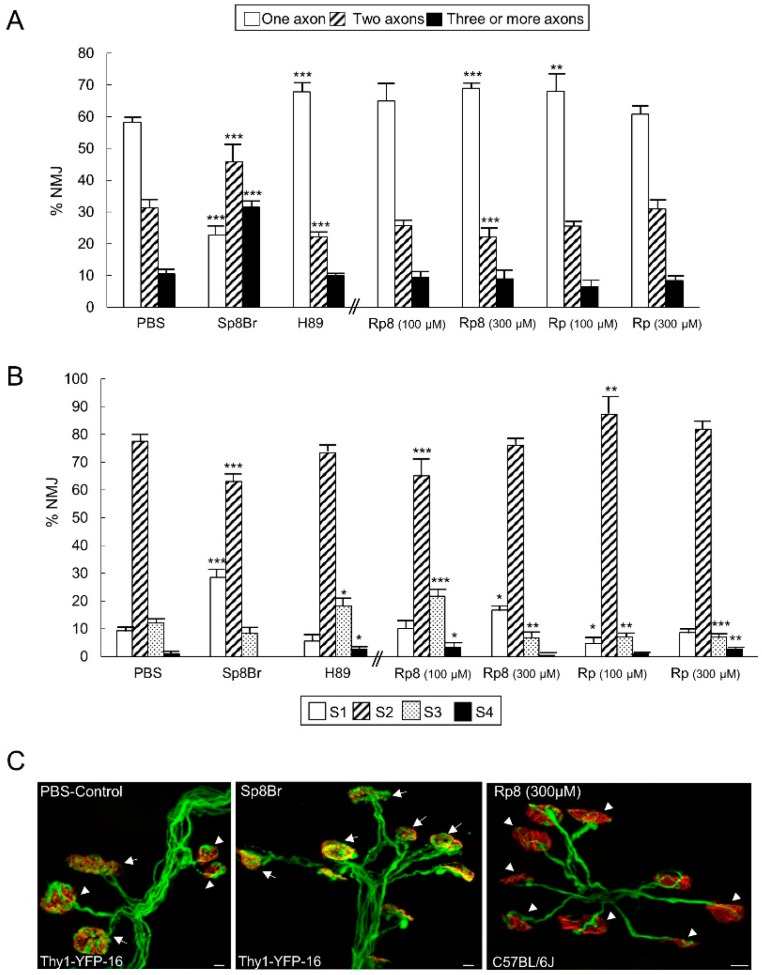
PKA activity modulation in axonal loss and postsynaptic receptor cluster maturation. The histogram in (**A**) shows the percentage of singly-, doubly-, and triply- (or more) innervated NMJs in the untreated control mice (only PBS) and after four applications (one application every day between P5 and P8, observation at P9) of one of the following substances: the PKA inhibitor H-89, the PKA stimulator Sp8Br, and the selective antagonists of the PKA regulatory subunits RI (Rp8) and RII (Rp) at two concentrations (100–300 μM). The data show that PKA inhibition accelerates axonal elimination while PKA stimulation delays axonal loss. The results on the selective antagonists of the RI and RII regulatory subunits suggest that some of the differences in the dose-dependence between the blocking peptides may be attributed to differences in affinity for the respective R subunits. The histogram in (**B**) shows that PKA inhibition moderately accelerates postsynaptic maturation while stimulation delays it. Thus, in normal conditions, PKA prevents developmental postsynaptic cluster differentiation. The results on the selective antagonists of the RI and RII subunits unexpectedly show that the RI inhibitor at 300 μM delays postsynaptic maturation, which suggests a downregulation effect at the highest dose of Rp8. Data were presented as percentages of NMJ ± SD (for each treatment and PBS control: *n* pups = 6–9; *n* = 11–18 *Levator auris longus* (LAL) muscles; *n* NMJ: PBS: 2538; H89: 1265; Sp8Br: 1324; Rp8 100 μM: 1140; Rp8 300 μM: 1287; Rp 100 μM: 1943; Rp 300 μM: 2102). Fisher’s test: * *p* < 0.05, ** *p* < 0.01, *** *p* < 0.005. The confocal images in (**C**) show representative areas of the LAL terminal innervation (control PBS, left; arrow heads in monoinnervated junctions and arrows in multiinnervated NMJ) in which PKA stimulation with Sp8Br (center) delays axon loss because many multiinnervated NMJs (arrows) persist. On the contrary, the antagonist of the PKA RI subunit (Rp8 at 300 μM, right) increases the number of monoinnervated junctions (arrow heads).

**Figure 3:**
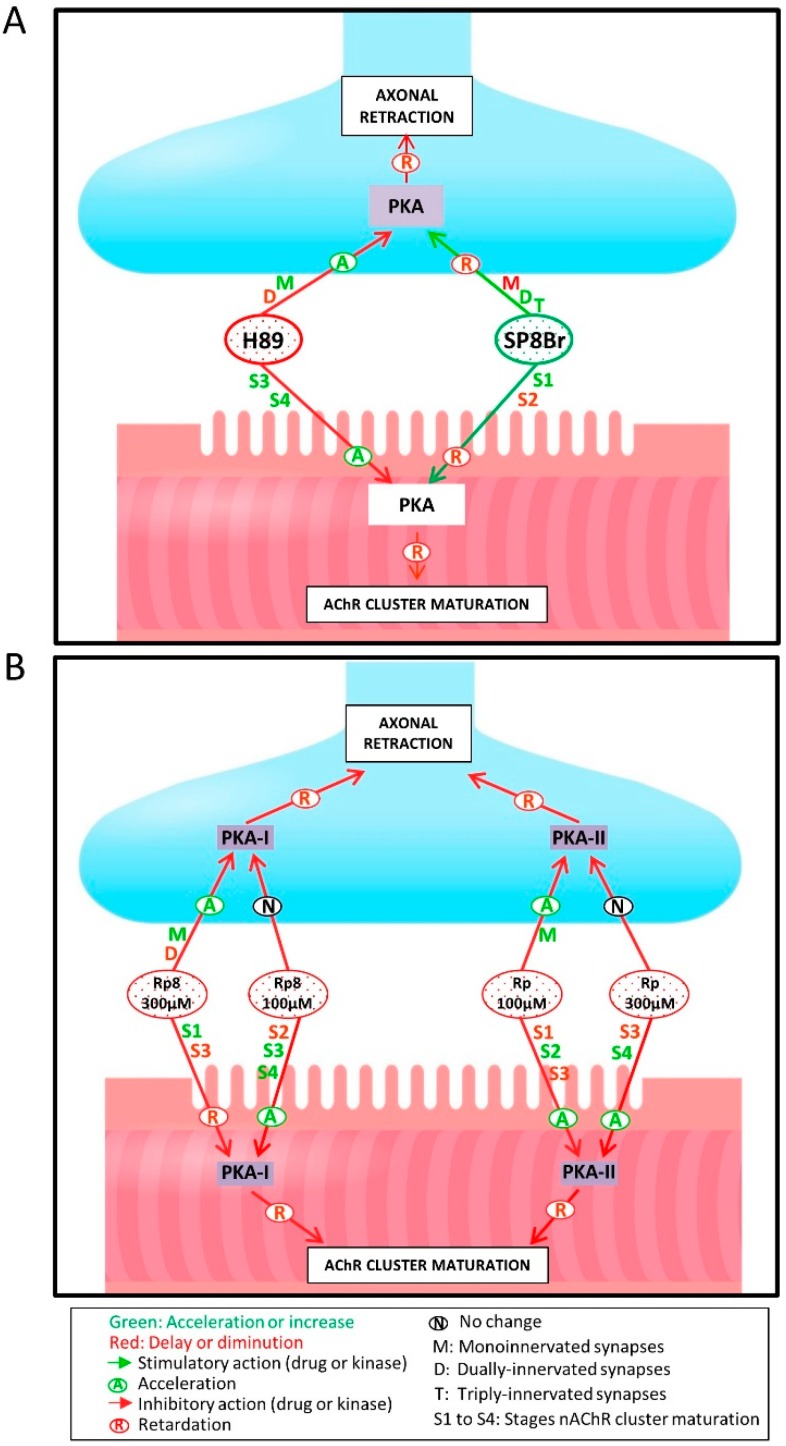
Diagrammatic representation of PKA involvement in pre- and postsynaptic NMJ maturation. (**A**) shows the effect on axonal retraction and nAChR cluster maturation of the unselective PKA inhibitor H-89 and stimulator Sp8Br. In (**B**), the selective antagonists of the regulatory PKA subunits RI (Rp8, 100–300 μM) and RII (Rp, 100–300 μM) discriminate the involvement of the PKA-I and PKA-II isozymes at the synaptic sites. Overall pre- and postsynaptic PKA activity coordinately prevents NMJ maturation by stabilizing the developmental situation.

Figure 2A shows the percentage of singly-, doubly-, and triply- (or more) innervated NMJs in the untreated control Thy1-YFP-16 expressing mice (only PBS) and after four applications (one application every day between P5 and P8; observation at P9) of one of the following substances: the PKA inhibitor H-89 (5 µM), the PKA stimulator Sp8Br (10 µM) (neither agent discriminates between the PKA subunits), and the selective antagonists of the RI (Rp8, 100–300 µM) and RII (Rp, 100–300 µM) PKA regulatory subunits. The data show that PKA inhibition (H-89) accelerated the rate of axonal elimination because of the significant increase in monoinnervated NMJs and the reduction in dual synapses, which led to a more rapid transition to the final monoinnervation state. On the contrary, PKA stimulation with Sp8Br considerably delayed axon loss because many doubly- (see Figure 2C center, arrows) and triply-innervated synapses persisted, so the percentage of monoinnervated junctions was around one third of the value expected at P9. Thus, exogenous stimulation revealed that the downstream PKA pathway had the potential to delay postnatal axonal disconnection, and this function was clearly observed by using the PKA inhibitor H-89, which revealed a tonic acceleration of axonal loss after PKA block (see also Figure 3A; the drawings in the Figure 3A,B, represent the complex results on pre- and postsynaptic synapse maturation after PKA stimulation and inhibition).

##### Specific Involvement of PKA Regulatory Subunits

We used the selective antagonists of the RI (Rp8) and RII (Rp) regulatory subunits of the PKA, which have an inhibitory influence on the catalytic subunits [32,46]. These antagonists prevent cAMP binding to the regulatory subunits thus blocking dissociation from the catalytic subunits and preventing them from acting. As expected, the data in Figure 2A indicate that the selective antagonist of the RI subunit (Rp8 at 300 μM but not at 100 μM) accelerates axonal loss because it significantly increases the number of monoinnervated junctions (see Figure 2C right, arrow heads) and, therefore, decrease the number of dually innervated junctions. The RII PKA regulatory subunit also seems to be involved in axonal stabilization because selective inhibition with Rp (in this case only at the lowest dose, 100 μM) accelerates axonal retraction (see also Figure 3B). 

#### 3.2.2. PKA Activity Prevents the Postsynaptic Receptor Cluster Maturation 

Figure 2B shows how the stimulation and inhibition of PKA affects the maturation of the postsynaptic nAChR clusters. The histogram shows the percentage of S1–S4 clusters in the NMJ of the untreated control mice (PBS) and after four applications of the PKA inhibitor H-89 or the PKA stimulator Sp8Br (between P5 and P9). Inhibiting PKA catalytic activity moderately accelerates postsynaptic maturation and increases the number of S3 and S4 clusters. Accordingly, stimulating PKA activity delays maturation because of the increased number of S1 clusters. The postsynaptic effect of both PKA agents coincides with the effect on presynaptic axon loss: namely, acceleration (H89) and delay (Sp8Br), respectively (Figure 3A). The results of using the selective antagonists of the RI and RII subunits show that in the postsynaptic site RII seems to be involved because Rp accelerates maturation (fewer S3 and more S4 clusters at 300 μM, and fewer S1 and more S2 at 100 μM). RI is also involved because the inhibitor Rp8 (at 100 μM) accelerates AChR cluster maturation (fewer S2 and more S3 and S4). Surprisingly, however, the RI inhibitor at 300 μM delays the maturation of the nAChR cluster because the number of S1 increases and the number of S3 and S4 decreases. This may indicate some downregulation of the PKA-I at the highest dose of Rp8. 

In summary, PKA activity seems to act at the pre- and postsynaptic sites by delaying both axonal elimination and nAChR cluster differentiation (see Figure 3).

### 3.3. PKC Activity Modulation

#### 3.3.1. PKC Activity Potentiates the Developmental Axonal Loss

**Figure 4:**
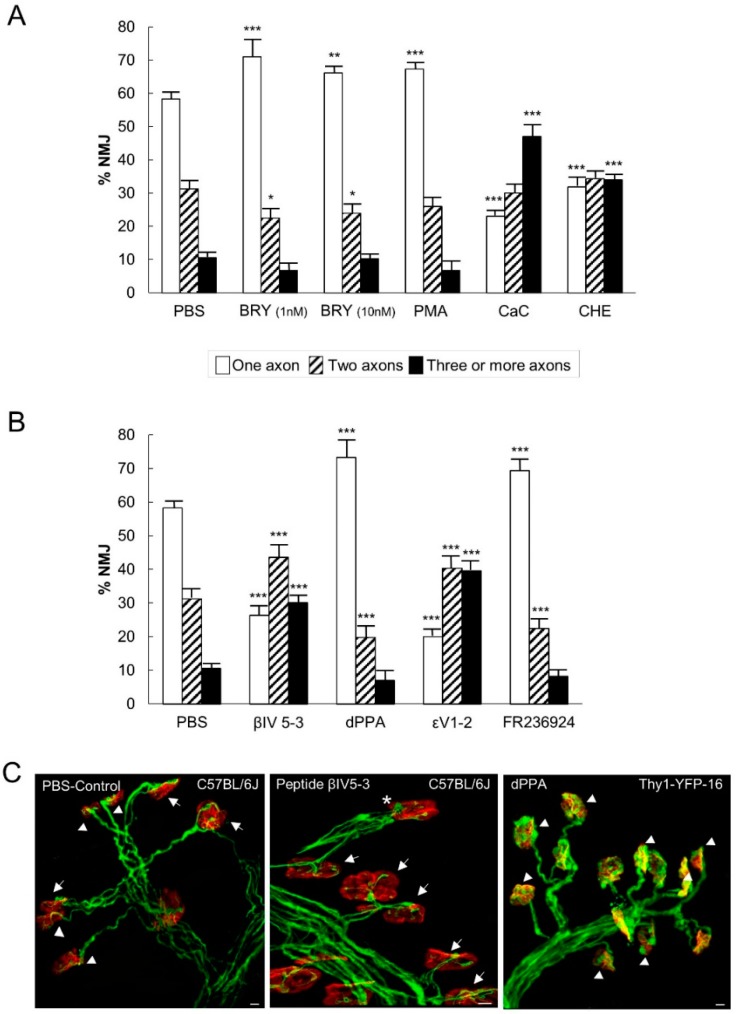
PKC activity modulation in axonal loss. (**A**) shows the percentage of singly-, doubly-, and triply- (or more) innervated NMJs in the control mice (PBS) and after four applications of one of the following substances: the PKC paninhibitors Chelerytrine (CHE) and CaC, and the PKC panstimulators (BRY, at 1 and 10 nM) and PMA. CHE and CaC action results in the persistence of many polyinnervated synapses. Accordingly, both PKC stimulators PMA and BRY increase the number of monoinnervated junctions and clearly decrease the percentage of doubly-innervated junctions in the case of BRY. (**B**) shows the percentage of singly-, doubly-, and triply (or more) innervated NMJs after exposure to the cPKCβI and nPKCε isoform selective inhibitors βIV_5–3_ and εV_1–2_, and the cPKCβI and nPKCε selective activators dPPA and FR 236,924 respectively. The selective inhibitors similarly increase the doubly- and triply-innervated synapses with the corresponding reduction in the monoinnervated junctions. The activators considerably accelerate nerve terminal elimination. Data were presented as percentages of NMJ ± SD (for each treatment and PBS control: *n* pups = 6–9; *n* = 11–18 LALs; *n* NMJ: PBS: 2538; Bry 1 nM: 1232 Bry 10 nM:1384; PMA:1247; CaC: 1573; Che:1499; βIV_5–3_: 1275; dPPA: 1121; εV_1–2_:1663 and FR 236924: 1367). Fisher’s test: * *p* < 0.05, ** *p* < 0.01, *** *p* < 0.005. The confocal images in (**C**) (control PBS, arrow heads in monoinnervated junctions and arrows in multiinnervated NMJ), show that the application of the inhibitor peptide βIV_5–3_ increases the number of multiinnervated NMJ (center, arrows) junctions, whereas the cPKCβI stimulator dPPA (but also the nPKCε selective activators FR 236924, not shown here) increases the number of monoinnervated junctions (right, arrow heads).

Figure 4A shows the percentage of singly-, doubly-, and triply- (or more) innervated NMJs in the control mice (PBS) and after four applications of one of the following substances: the PKC pan-inhibitors Chelerytrine (CHE, 1 µM) and Calphostin C (CaC, 200 nM), the PKC stimulators Bryostatine-1 (BRY, 1–10 nM), and a phorbol ester (PMA, 10 nM). These agents do not discriminate the different PKC isoforms. Exposing the LAL muscle to the agent CHE, which inhibits the C3 catalytic active site of all the PKC isoforms, considerably reduced the number of monoinnervated synapses with the persistence of many triply-innervated synapses and, therefore, delayed the axonal elimination process. A similar result was observed by using the PKC paninhibitor CaC, which acts on the regulatory domain C1. This suggests that PKC is tonically involved in promoting axonal loss (see also the summarized results in Figure 6A). Accordingly, the PKC stimulators PMA and BRY (acting on the C1 domain) accelerated axonal elimination by increasing the number of monoinnervated junctions and decreasing the percentage of doubly-innervated junctions in the case of BRY (at the two doses used 1 and 10 nM). Interestingly however, we assayed higher doses of PMA and observed the well characterized effect of PKC downregulation. Specifically, 100 nM PMA even delayed axon loss because the percentage of polyinnervated synapses increased (data not shown because a similar result was previously published [30]). PKC activity coordinately promoted both pre- and postsynaptic maturation. 

##### Specific Involvement of cPKCβI and nPKCε Isoforms 

By using immunohistochemistry, these isoforms were located in the nerve terminal of the NMJ, regulated by synaptic activity, and involved in neurotransmitter release in the adult [39,40,45,47]. Thus, they are good candidates for being involved in developmental axonal plasticity. To investigate this, between P5 and P9, LAL muscles were exposed to cPKCβI and nPKCε isoform selective inhibitors, the cPKCβI-specific translocation inhibitor peptide βIV_5–3_ (10 µM, [48]), the nPKCε-specific translocation inhibitor peptide εV_1–2_ (100 µM [49]), and finally, the cPKCβI and nPKCε highly selective activators dPPA (0,2 µg/mL) and FR 236,924 (100 nM), respectively.

We previously demonstrated that εV_1–2_ and βIV_5–3_ peptides inhibit the presence of phospho-nPKCε and phospho-cPKCβI, respectively, in the synaptic membrane [39,40,47] and thus inhibit the phosphorylating activity of these isoforms on several presynaptic proteins related with neurotransmission (for instance Munc18-1, SNAP-25, and MARKS) [40,50,51]. Figure 4B (see also Figure 6B) shows that the application of both inhibitor peptides significantly increased the number of doubly- and triply-innervated synapses and correspondingly reduced the monoinnervated junctions, thus considerably delaying the axonal elimination process (see also the Figure 4C center, arrows). Interestingly, the selective block of the PKCε or β1 resulted in an important similar retention of the triply-innervated synapses suggesting that the PKC-mediated mechanism of axonal disconnection operated from the beginning of the postnatal period and axonal competition (when NMJs were highly polyinnervated). This delay was comparable to that observed when the PKC pan-inhibitors CHE and CaC (see Figure 4A) were used. 

Due to the major role of the PKCβ1 and PKCε, we selectively stimulated these isoforms with dPPA or with FR 236924, respectively [35,37]. Figure 4B shows that the preferential stimulation of cPKCβI with dPPA accelerated axonal loss (it increased the number of monoinnervated junctions and reduced the number of dual junctions (see the Figure 4C right, arrow heads). A similar situation was observed when PKCε was stimulated with FR 236924. These results were also similar to the effect of BRY and PMA (Figure 4A). 

In summary, these data indicated that i) blocking each isoform delayed axonal elimination and therefore NMJ maturation—observed at P9—to a similar extent (the percentage of monoinnervated junctions was only 20%, the same as at the onset of the experiment at P5 (see also [17]) compared to the 60% observed at P9 in untreated muscles), so the two isoforms played a similar role and both contributed to developmental axon loss, ii) blocking each isoform had an effect that was similar to unselectively blocking all isoforms with CHE (or CaC), which showed that each isoform had the full PKC effect on axon loss, and iii) blocking each isoform had an effect that was similar to that of PKA stimulation, which strongly suggests that the balance between these kinases may play a role in synapse elimination (see Figure 6A).

#### 3.3.2. PKC Activity Promotes the Postsynaptic Receptor Cluster During Postnatal Maturation

**Figure 5:**
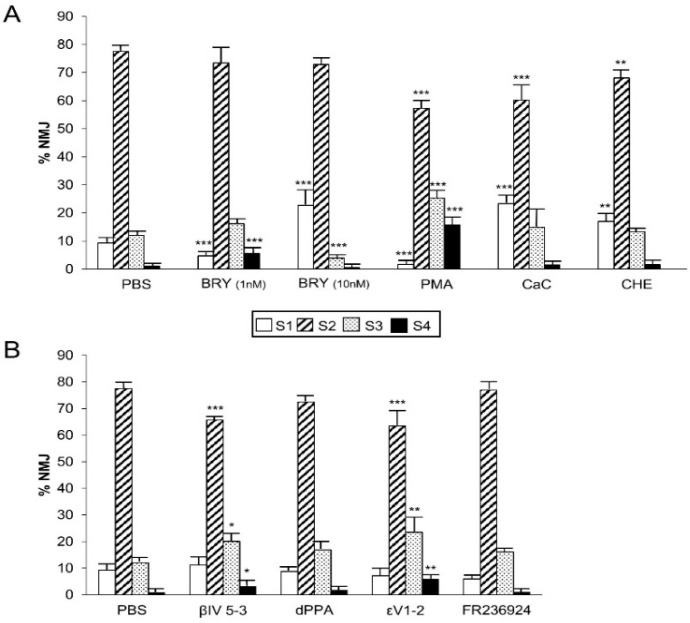
Stimulation and inhibition of PKC modifies the postsynaptic receptor cluster during postnatal maturation. The histogram in (**A**) shows that the unselective stimulation and inhibition of PKC affects the maturation of the postsynaptic nAChR clusters. Inhibition with CHE or with CaC results in the persistence of the immature S1 clusters. Accordingly, PMA (10 nM) accelerates postsynaptic maturation and a similar effect is observed with BRY (1 nM). However, 10 nM BRY downregulates the PKC at the postsynaptic site. (**B**) shows that the specific stimulation and inhibition of cPKCβI and nPKCε isoforms affects the maturation of the nAChR clusters. Blocking these kinases accelerates postsynaptic maturation though the selective stimulation with dPPA and FR 236,924 respectively show no influence on the maturation of the nAChR clusters. Data were presented as mean ± SD (for each treatment and PBS control: *n* pups = 6; *n* = 12 LALs). Fisher’s test: * *p* < 0.05, ** *p* < 0.01, *** *p* < 0.005.

**Figure 6:**
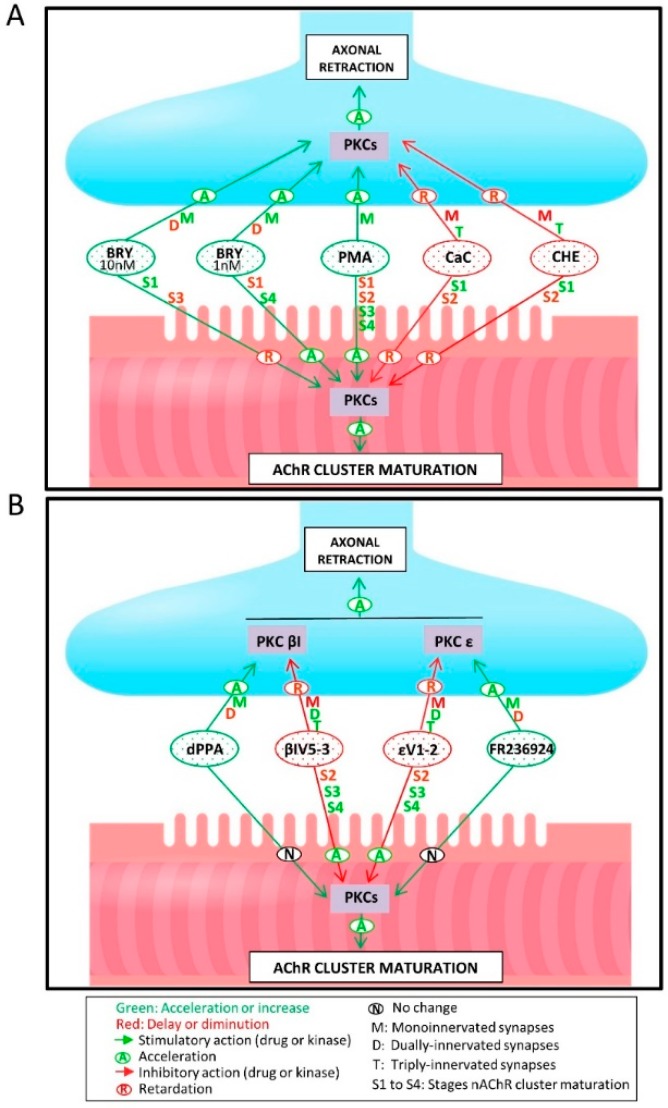
Diagrammatic representation of PKC involvement in synapse maturation. (**A**) shows the effect on axonal retraction and nAChR cluster maturation of the unselective PKC inhibitors and PKC stimulators. (**B**) shows the effect of the nPKCε and cPKCβI (modulators).

Figure 5A (see also Figure 6A) shows that PKC inhibition with CHE or with CaC delayed the maturation of the postsynaptic apparatus because the most immature S1 clusters persisted to some extent. This delay was in agreement with the presynaptic delay in axon loss produced by these substances. Accordingly, PMA (10 nM) accelerated postsynaptic cluster maturation by reducing S1 and S2 clusters and increasing the most mature S3 and S4 clusters. A similar effect was observed with BRY (1 nM). As with the effect of high doses of PMA on axon loss, we found here that the high dose of BRY (10 nM) may downregulate the PKC at the postsynaptic site because of the delay in the postsynaptic maturation observed (the persistence of many S1 and the reduction in S3 clusters).

We next investigated the specific involvement of the cPKCβI and nPKCε isoforms in postsynaptic AChR cluster maturation. Figure 5B shows that blocking the nPKCε accelerated postsynaptic maturation by notably increasing the number of S3 and S4 clusters. Likewise, blocking the cPKCβI isoform increased the number of S3 clusters. The selective stimulation of these isoforms with dPPA and FR 236924, respectively, had no influence on the maturation of the nAChR clusters.

### 3.4. Comparison Between the PKA and the PKC Pathways 

We compared the PKC and PKA activity modulation of the axonal loss and postsynaptic receptor cluster maturation. First, we compared all results after PKC stimulation with the results after PKA inhibition and reciprocally (the highest doses of the analyzed substances were not included in these comparisons). As expected, a non significant difference in the percentage of monoinnervated junctions (as example of axon loss) and in the percentage of the most immature nAChR clusters S1 or S2 (as an example of postsynaptic maturation) was observed (in all cases *p* > 0.05; data not show). Thus, when a kinase was activated and the other was inhibited, no significant difference could be observed in the synapse maturation parameters in all cases. Second, we compared the results obtained after stimulation (or inhibition) of both kinases. This comparison showed a significant difference in relation with the percentage of the monoinnervated NMJ in all paired comparisons (*p* < 0.05) and in the majority of the comparisons (more than two thirds) in relation with the percentage of the immature nAChR clusters. These results strongly indicate that these kinases have almost exactly opposed effects.

### 3.5. Prolonged Inhibition of PKA and PKC

To investigate the effect of the persistent block of the PKA-I and cPKCβI (molecules selected as a representative example) on axon loss throughout the period of synapse elimination, we made daily applications of their blockers between P5 and P15 (90% of NMJs were monoinnervated at P15). In spite of the continued inhibition, the axonal elimination process ended normally by the end of the second postnatal week (PBS: *n* = 6 mice, 12 muscles, and 1543 NMJs of which 1418 (91.9% ± 4.31) were monoinnervated, 86 (5.57% ± 1.76) dually innervated, and 39 (2.53% ± 0.85) triply innervated; for βIV_5–3_: *n* = 6 mice, 12 muscles, and 1320 NMJs of which 1187 (89.88% ± 5.86) were monoinnervated, 106 (8.03% ± 1.36) dually, and 27 (2.09% ± 0.87) triply innervated; for Rp8 (300 µM): *n* = 6 mice, 12 muscles, and 1403 NMJs of which 1317 (93.86% ± 6.29) were monoinnervated, 61 (4.34% ± 0.99) dually, and 25 (1.8% ± 0.53) triply innervated; *p* > 0,05). We conclude that a PKA–PKC mechanism modulates the conditions of the competition between nerve endings, but the final process of axonal disconnection can be differentially regulated.

## 4. Discussion

During the histogenesis of the nervous system, synaptic contacts are produced in excess. Signaling through membrane receptors between the cells that make the synapses allow activity-dependent consolidation of the best functioning connections and the elimination of the redundant or inappropriate ones [14,52,53]. In the NMJ, activity dependent variations of released mediators such as Ach, adenosine, and neurotrophins are sensed by presynaptic mAChR, AR (P1), and the TrkB receptors [17,54,55], as well as ATP receptors (P2) and glutamate receptors [18,19,56,57]. These receptors are coupled to downstream kinases, mainly PKC and PKA, that phosphorylate the protein targets involved in axonal disconnection or consolidation and synapse maturation [29,58,59]. 

### 4.1. PKA and NMJ Maturation

We found that PKA activity tends to stabilize the polyinnervated postnatal junctions. PKA is present in a variety of cells, is involved in many different pathways, and acts on different substrates, so they are difficult to regulate [60,61]. The regulatory subunits of PKA exist in two isoforms, RI and RII, which distinguish the PKA isozymes, type I (PKA-I) and type II (PKA-II). Experimental evidence has shown that the selective modulation of the two isoforms of PKA may act respectively as positive and negative intracellular regulators in some cases [32,62]. However, we observed that both PKA-I and PKA-II isozymes are similarly involved in pre- and postsynaptic sites. Both localizations stabilize the current situation. However, the RI inhibitor at the highest concentration (300 μM) delays the postsynaptic maturation (contrary to what may be expected). This suggests that the RI regulatory subunit may be downregulated at the highest dose of Rp8, similarly to what occurs with the highest concentrations of PMA and BRY in relation with PKC activity and effect.

#### 4.1.1. PKA in the Presynaptic Site

PKA in the axon terminals is directly involved in tonically modulating calcium-dependent ACh release [33,38]. PKA stimulation increases transmitter release in many cells [63] mainly by their action on the P-type voltage gated calcium channel (VGCC), and, in the adult, PKA activity promotes PKC activity [38,64] and is functionally associated with mAChR [54,65], neurotrophin receptors [66,67], and AR signaling [22]. However, differences in PKA coupling to ACh release in each of the different axon terminals in competition at the same synaptic site during development had not been investigated yet (this information is available for PKC, see later). During synapse elimination, PKA activity may potentiate transmitter release even in the less favored axons, thus delaying their activity-dependent competitive elimination. One of the main regulators of PKA is M_2_ signaling, which blocks adenyl cyclase and PKA activity [20,21]. Thus, as expected, we previously found that the M_2_ pathway accelerates axon loss between P5 and P9 (a tonic effect observed when blocked with methoctramine [16,17]). 

Another regulator of PKA is A_2A_ adenosine receptor signaling, which has the opposite effect to M_2_ signaling. It stimulates adenyl cyclase and PKA activity [29,59,68]. Thus, the A_2A_-mediated increased PKA activity can be expected to cause a delay in axon loss similar to the one observed here when PKA was experimentally directly activated. We clearly observed this effect at P7 [17], although at P9 both A_2A_ and M_2_ signaling actually resulted in accelerating axonal loss. Altogether, this shows the complexity of the PKA involvement in synapse elimination and indicates that the process is also controlled by other mechanisms as the PKC-based pathway. The prevalence of one pathway over the other is dependent on membrane-bound receptor activation, which varies significantly with the pattern of nerve activation. The crosstalk between AC/PKA and PLC/PKC/IP3 at the NJM must be take into account. It has been proposed that PKA phosphorylation of SNAP-25 at Thr-138 controls the size of the releasable vesicle pools, whereas PKC phosphorylation of SNAP-25 at Ser-187 is involved in regulating refilling after the pools have been emptied [69,70]. Thus, PKA activity may have a stabilizing effect by modulating the size of vesicle pools in the competing nerve endings.

#### 4.1.2. PKA in the Postsynaptic Site 

We found that PKA activity delays both the morphological maturation of the nAChR cluster and axon loss. PKA activity delays the S1–S2 differentiation to the more structured pattern of independent primary gutters (S4). It is known that the activation of PKA and PKC has opposite effects on nAChR stability in the membrane [71]. PKC activity increases the phosphorylation of the nAChR delta (and decreases receptor stability) while PKA activity modulated by the RIα subunit, increases the phosphorylation of the epsilon subunit (and increases receptor stability) [64,72,73,74]. It has been proposed that synaptic activity-dependent postsynaptic PKA activation (mediated by presynaptically released peptides such as CGRP) stabilizes the nAChR at pre–post synaptic appositions. In addition, in the present experiments of PKA activation, the persistence of several active nerve terminals would contribute to the stabilization and persistence of the uniform S1–S2 nAChR clusters in the postsynaptic site.

### 4.2. PKC and NMJ Maturation

Despite the pre- and postsynaptic stabilizing effect of PKA during NMJ development that prevents maturation, these synapses progressively differentiate the pre- and postsynaptic sites to become monoinnervated over high-density nAChR postsynaptic pretzel-like gutters. We found here that PKC activity tends to accelerate the supernumerary axonal elimination and nAChR cluster maturation (Figure 6). 

#### 4.2.1. PKC in the Presynaptic Component

PKC activity enhances ACh release in all synaptic contacts in multiinervated junctions, regardless of their developmental maturation, except in the weaker synaptic contact during axonal competition [33,54]. So, the weakest nerve terminal is potentiated by PKC block and some silent nerve endings even recover neurotransmitter release in this condition [75]. In addition, we found that reducing calcium entry through P/Q-, N-, or L-type VGCC—all channels are present in motor axons during development—or even by selectively blocking the M_1_-type mAChR [38,54,55], all of which are major upstream inductors of PKC activation, has the same effect as the kinase block. The developmental M_1_ coupling to ACh release and axonal loss clearly involves a specific PKC and VGCC adaptation in some nerve terminals, which leads to differences in transmitter release and presumably competitive force between the axon terminals.

We do not know how the PKC signaling changes in the competing nerve endings but here we found that selectively blocking cPKCβI and nPKCε isoforms delays axonal loss. This is probably due to the increased transmitter release and persistence of the weakest axons in these conditions. We observed that blocking cPKCβI (calcium-dependent) and nPKCε (calcium-independent) isoforms [39,58], delays axonal loss to a similar extent as the unselectively block of all isoforms. It seems that there is a common downstream pathway for PKCε and PKCβ1 in terms of developmental synapse elimination. Their target may be the same molecule or a different one on the same pathway, but the two isoforms are necessary, sufficient and interdependent for PKC involvement in axon loss. If one of them is blocked, the other cannot substitute it. Our hypothesis is that the first isoform has a priming effect that allows the second one to intervene. Moreover, we recently found that both cPKCβI and nPKCε are involved in Munc18-1 phosphorylation [51] while only nPKCε intervenes in SNAP-25 phosphorylation during synaptic activity in the adult [50]. 

The downstream links of receptors other than the M_1_ receptor with PKC seem to contribute to synapse elimination. In fact, A_1_ and TrkB also change the axonal elimination rate between P5–P9 [16,17,76]. Neurotrophins and their receptors show different spatial and temporal expression during NMJ development [77,78,79,80,81]. Brain-derived neurotrophic factor (BDNF) increases ACh release in all synaptic contacts, regardless of their developmental maturation. Thus, the TrkB effect favoring axonal loss at P9 may be related to the combined effect of PKC-mediated transmitter release potentiation of some nerve terminals with differences in BDNF availability and signaling between the axons that are in competition (the most active may be differentially rewarded by postsynaptic-derived BDNF). 

#### 4.2.2. PKC in the Postsynaptic Component

Here we found that PKC activity accelerates the differentiation of the nAChR cluster and stimulates axon loss. During synapse elimination, some presynaptic inputs become less efficient because a progressively smaller quantal content is associated with a decreased density of postsynaptic nAChRs [82]. Thus, the maturation of the NMJ transforms the nAChR clusters at the postsynaptic site. PKC activity disperses the nAChRs and accelerates the morphological maturation of the receptor clusters [44,83,84,85].

The activation of PKA and PKC has opposite effects on nAChR stability in the membrane [71] associated to changes in the phosphorylation of different receptor subunits [73,74]. The PKA/PKC interaction could sharpen areas of highly concentrated, stabilized nAChR (dominated by PKA phosphorylated receptors at pre-post synaptic appositions) and receptor-poor areas in which PKC (in the absence of PKA action) has destabilized the receptor [58].

The nPKCθ isoform might affect the phosphorylation and stability of the receptor clusters. This isoform is strongly expressed in muscle under neural control mainly during development [86,87], inhibits the receptor clustering function of agrin [88], and in knockout nPKCθ mice the process of synapse elimination is affected [87]. It seems that the target of PKCθ phosphorylation at the postsynaptic site is the delta subunit of the nAChR [73].

Interestingly, cPKCβI and nPKCε isoforms are undetectable at the postsynaptic site of the NMJ [40,58]. This is in agreement with the observation that the selective stimulation of these isoforms has no influence on the maturation of the nAChR clusters (Figure 6B). However, selectively blocking these isoforms accelerates postsynaptic maturation with a notable increase in the more mature S3 and S4 clusters. This result indicates that in normal conditions (without ε and βI block) these isoforms can contribute in some way to delaying postsynaptic cluster maturation. The observed postsynaptic effect may be indirectly produced as a consequence of their action on axonal competition and loss. The result observed after selectively blocking cPKCβI and nPKCε is the retention of polyinnervation and the acceleration of AChR cluster maturation. This suggests that there is some sort of inverse relation between the presynaptic effect on axonal loss rate (diminution) and the postsynaptic receptor cluster morphological maturation (acceleration). In fact, these processes take a different course when several receptor pathways in the synaptic signaling are blocked [16,17,43,44,89]. One plausible explanation for this is that postsynaptic differentiation depends more on a strong presynaptic function than on the number of inputs. The retention of axons after the PKC block may reinforce more than one axon and lengthen the competition between the strongest endings. The activity of these endings at the postsynaptic site may influence postsynaptic maturation.

Moreover, when all PKC isoforms are blocked with the paninhibitors CHE and CaC, cluster maturation is delayed, indicating that the tonic activity of some postsynaptic isoform/s, other than nPKCε and cPKCβI, promotes postsynaptic maturation. When all isoforms are blocked with paninhibitors like CHE, the positive effect of strong axon retention on nAChR maturation seems to be canceled out by the simultaneous block of the postsynaptic PKC isoform.

Interestingly, the PKC stimulator BRY has a curious dose-dependent effect on the postsynaptic site. As expected, BRY (1–10 nM) accelerates axonal loss and, therefore, decreases the number of axons at the observation time. Postsynaptic maturation would also be expected to accelerate as a result of the stimulation of the postsynaptic isoform and this effect is observed at the lowest BRY dose (1 nM). However, the opposite effect is observed at 10 nM BRY which clearly delays nAChR cluster maturation. We believe that a downregulation effect similar to that observed at the higher doses of PMA is produced at the high dose of BRY [30].

We extended until P15 the direct inhibition of cPKCβI and PKA-I. Our data show that the elimination process comes to the normal conclusion at P15 in both situations, similarly as occurs after the extended inhibition of several presynaptic membrane receptors (mAChR, AR and TrkB) coupled to these kinases [17]. Thus, the described PKC-PKA mediated mechanism of axon loss modulates the conditions of the competition between nerve endings, possibly helping to determine the winner or the loser but the final synapse elimination would occur with some autonomy.

## 5. Conclusions

Despite the stabilizing effect of PKA during NMJ development, the tendency to mature dominates, and, thus, the PKC activity seems to be the determining factor. These kinases have almost exactly opposed effects. We hypothesize that the prevalence of a PKC-mediated mechanism in some endings contributes to their destabilization and retraction. Moreover, the prevalence of one pathway over the other may be regulated by presynaptic membrane receptors activation (mAChR, AR, and/or TrkB receptors), which vary with the pattern of nerve activation. An activity-dependent displacement of the PKA/PKC activity ratio to lower values in, both, nerve endings and postsynaptic sites have a role in developmental synapse elimination. Here we found that PKA prevents developmental axonal elimination while the PKC activity promotes it, as cPKCβI and nPKCε isoforms are both necessary, sufficient, and interdependent for PKC involvement in axon loss. The first intervening PKC isoform may have a priming effect that allows the second kinase to intervene. Blocking a PKC isoform has an effect that is similar to PKA stimulation, which suggests that the balance between PKA and PKC is essential in synapse elimination. PKA-I and II isozymes coordinately contribute to delaying both axonal elimination and nAChR cluster differentiation. PKC activity promotes both axonal loss and postsynaptic maturation (with the involvement of the PKCθ). Finally, we found indications of a prevalence of the presynaptic change in axon number and activity over postsynaptic differentiation. These results provide greater insight into the mechanisms involved in synaptic formation and maintenance, which are affected in several diseases.

## Abbreviations

ACh: acetylcholine; AC, adenyl cyclase; AKAP, A-kinase anchor protein; AR, adenosine receptors; A_1_, adenosine receptor; A_2A_, adenosine receptor; BDNF, brain-derived neurotrophic factor; BRY, bryostatine-1; CaC, calphostin C; Che, chelerythrine; P3, the inositol triphosphate; LAL, Levator auris longus muscle; mAChR, muscarinic acetylcholine receptor; MARCKS, myristoylated alanine-rich C-kinase substrate; MS, morphological stage; nAChR, nicotinic acetylcholine receptor; M_1_, M_1_-type muscarinic acetylcholine receptor; M_2_, M_2_-type muscarinic acetylcholine receptor; M_4_, M_4_-type muscarinic acetylcholine receptor; Munc18, mammalian uncoordinated-18; NMJ, neuromuscular junction; PBS, phosphate buffer saline; PKA, protein kinase A; PKA-I, PKA isozymes type I; PKA-II, PKA isozymes type II; PKC, protein kinase C; cPKCβI, conventional protein kinase C beta I; βIV_5–3_, cPKCβI specific translocation inhibitor peptide; nPKCε, novel protein kinase C epsilon; εV_1–2_, nPKCε, specific translocation inhibitor peptide; PKCθ, protein kinase C theta; PLC, phospholipase C; PMA, phorbol 12-myristate 13-acetate; Rp, Adenosine-3′,5′-cyclic monophosphorothioate, Rp-isomer sodium salt; Rp8, 8-Bromoadenosine-3′,5′-cyclic monophosphorothioate, Rp-isomer sodium salt; SNAP-25, synaptosomal nerve-associated protein 25; TRITC-α-BTX tetramethylrhodamine α-bungarotoxin; TrkB, tropomyosin-related kinase B receptor; VGCC, voltage gated calcium channels.
